# Detection of coagulase gene in *Staphylococcus aureus* from several dairy farms in East Java, Indonesia, by polymerase chain reaction

**DOI:** 10.14202/vetworld.2019.68-71

**Published:** 2019-01-10

**Authors:** Mustofa Helmi Effendi, Mirza Atikah Madarina Hisyam, Poedji Hastutiek, Wiwiek Tyasningsih

**Affiliations:** 1 Department of Veterinary Public Health, Faculty of Veterinary Medicine, Airlangga University, Surabaya, Indonesia; 2 Department of Veterinary Parasitology, Faculty of Veterinary Medicine, Airlangga University, Surabaya, Indonesia; 3 Department of Veterinary Microbiology, Faculty of Veterinary Medicine, Airlangga University, Surabaya, Indonesia

**Keywords:** coagulase gene, coagulase test, polymorphism, raw milk, *Staphylococcus aureus*

## Abstract

**Aim:**

This study was conducted to study the coagulase (*coa*) gene-based genetic diversity of *Staphylococcus aureus*, isolated from different samples of cattle from three different regions in East Java Province, Indonesia.

**Materials and Methods:**

A total of 160 raw milk samples collected in East Java Province, Indonesia, were screened for the presence of *S. aureus*. The presumptive isolates were confirmed by *coa* test. The confirmed *S. aureus* isolates were subjected to *coa* gene polymerase chain reaction.

**Results:**

Of 160 different samples, 20 (12.5%) isolates of *S. aureus* were confirmed by positive *coa* test. Of 20 *S. aureus* isolates, 19 (95%) isolates carried *coa* gene. Six different genotypes of *coa* gene, i.e., 440 bp, 510 bp, 547 bp, 680 bp, 740 bp, and 820 bp were obtained. One *coa* genotypes, 510 bp (10 isolates) were observed in polymorphism to be more prevalent than the others, and the genotype was present in at least one isolates from every region.

**Conclusion:**

It can be concluded that *coa* gene is easily epidemiological tool for detection of variation strain from *S. aureus*.

## Introduction

*Staphylococcus aureus* is the most pathogenic bacteria species of the genus *Staphylococcus* [1]. *S. aureus* can be isolated from domestic and food animals and associated with disease such as mastitis [2]. *S. aureus* secretes two clotting factors, coagulase (*coa*) protein and von Willebrand factor binding protein [3]. *coa* protein is an important phenotypic determinant and virulence factor of *S. aureus* [4]. The ability of its *coa* to clot plasma is a defining property of *S. aureus* and distinguished the species from other *coa*-negative staphylococci [5].

Variable genome structure that is associated with strains variant in the certain area shown by *S. aureus* was known to be responsible for the emergence of different epidemiological profiles [6]. Staphylocoagulase, as the major phenotypic determinant of *S. aureus*, exists in various allelic forms caused by the genetic variance in its 3’-end coding region [7]. The variations in its 3’ region have resulted in the gene to have polymorphic properties which, therefore, the same analysis result in all strains would not be possible [8]. The distinguishing factor of *S. aureus* *coa* gene lies in the heterogeneity of the region containing multiple repeated strands with 81 bp length in the 3’ region of the gene. Each *S. aureus* strains have differences in replication number and gene restriction location [9]. Polymerase chain reaction (PCR) amplification results of this region showed different size and number of DNA bands which can be differentiated further using enzyme restriction [7]. The unique property of staphylocoagulase which can be easily analyzed using a simple technique such as PCR amplification and the availability of this enzyme in all strains of *S. aureus* made *coa* gene amplification to be the simplest molecular typing method in *S. aureus* epidemiological study. Using this epidemiological method, *coa* gene typing is considered a simple and effective method for typing *S. aureus* isolates from bovine mastitic milk [10]. Epidemiological studies based on analysis of the *coa* gene have shown that *S. aureus* isolates could be divided into a number of subtypes, but only a few are responsible for most cases of bovine mastitis in different geographical areas [11].

In Indonesia, especially in East Java, little is known about the genotypic variance and the distribution of *S. aureus* isolated from raw cow’s milk. Therefore, the aim of this study was to detect the genotype variance of *S. aureus* isolated from raw cow’s milk sample in three regions in East Java based on its *coa* gene by PCR amplification and to understand the strains distribution.

## Materials and Methods

### Ethical approval

Raw milk were used in this study, hence ethical approval was not necessary. Raw milk samples were collected from three regions in East Java province, Indonesia.

### Bacterial isolates

A total of 20 *S. aureus* isolates from raw milk obtained from several farms in three regions such as Pasuruan region for Nongkojajar and Grati farm, Malang region for Batu farm, and Lumajang region for Senduro farm in East Java, Indonesia, were used in this study that shown in Table-1. The isolation and identification were performed for counting bacteria using conventional phenotyping method involved mannitol salt phenol red agar growth (E. Merck, Darmstadt, Germany), Gram staining, microscopic observation, catalase test, and tube *coa* test [12].

**Table-1:** Number of raw milk samples and *Staphylococcus aureus* from several dairy farms.

Name of farm	Number of samples	Positive *Staphylococcus aureus*
Nongkojajar (P)	32	9
Grati (G)	43	4
Batu (B)	49	3
Senduro (S)	36	4
Total	160	20

### DNA preparation

All *S. aureus* isolates were subcultured on MSA and incubated at 37°C for 24 h before DNA extraction. The DNA of all *S. aureus* isolates in this study was extracted using QIAamp^®^ DNA Mini Kit (QIAGEN, Singapore) and done using the manufacturer method.

### PCR amplification of the coa gene

For PCR amplification, a total of 50 µl reaction mixture contained 28 µl Go taq green master mix (Promega, Germany), 20 µl RNase free water, and 1 µl of each forward and reverse primer was prepared. The primer used for *coa* gene amplification as described by Hookey *et al*. [13] was 5’ATA GAG ATG CTG GTA CAG G3’and 5’GCT TCC GAT TGT TCG ATG C3’. A total of 2.5µl of DNA template were added to the mixture. The mixture then amplified using PCR cycler according to the protocol of Akineden *et al*. [14] with modification as following: Predenaturation at 94°C for 45 s, followed by 30 cycles of denaturation at 94°C for 1 min, annealing at 58°C for 1 min, and extension at 72°C for 1 min. The amplification ended by a final extension at 72°C for 2 min. The presence of PCR products was determined by electrophoresis of 10 µl of products in 2% agarose gel with TBE buffer and 100 bp DNA ladder as a marker (Promega, Germany).

## Results

**Figure-1:**
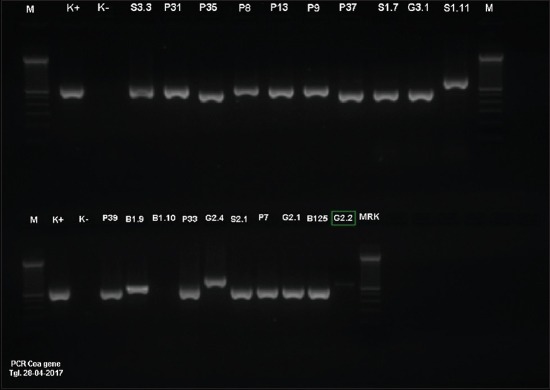
Agarose gel electrophoresis of *Staphylococcus aureus* polymerase chain reaction products. Lane M=1: 100 bp molecular weight standard, K+=Control positive, K-=Control negative, Senduro farm (S), Nongkojajar farm (P), Batu farm (B), Grati farm (G).

The 19 *S. aureus* isolates produced a single band with variance molecular size ranging from 440 bp to 820 bp (Figure-1). One isolate in this study did not produce the band. The 20% of isolates accounted for 4 of 20 produced a single band of 440 bp length. A single band with a molecular size of 510 bp was produced by most (50%) of the isolates. A single band with 547 bp, 680 bp, and 820 bp length was produced by one (5%) isolate, respectively. The 10% (2/20) isolates produced single band with molecular size 740 bp. According to the size of the product, the *S. aureus* isolates in this study can be grouped into 7 groups (Table-2).

**Table-2:** Group of isolates based on *coa* gene amplification product size.

Groups	Molecular size (bp)	Number of isolates	Percentage
Group A	440	4/20	20
Group B	510	10/20	50
Group C	547	1/20	5
Group D	680	1/20	5
Group E	740	2/20	10
Group F	820	1/20	5
Group G	No band	1/20	5

*coa*=Coagulase

## Discussion

In this study, 20 *S. aureus* strains were subtyped by *coa* gene PCR and resulted in single amplicon which indicates no size polymorphisms [15]. Amplification of *coa* gene showed six different PCR products of 440, 510, 547, 680, 740, and 820 bp. The difference of amplification products reflects the variation in *coa* gene length among *S. aureus* strains. Former studies were done by other researchers [13-20] using the same primer pairs and also showed that different *coa* gene types exist. The reason behind this polymorphism is unclear, but it is likely caused by the insertion, or deletion mutations of some portions in 3’ end region of *coa* gene resulted in a change of the gene size and probably also the antigenic properties of the enzyme. This region of the gene may have an important role in antigenic variation and its defense some inhibitory mechanism of anti-*coa* agents [18].

The *coa* gene PCR amplification of 20 *S. aureus* isolates revealed 6 *coa* PCR types. The most prevalence (50%) *coa* gene type was 510 bp length which found in at least one isolate of *S. aureus* from every location. Studies [16,18,21,22] from different countries showed that various *coa* types can be found in *S. aureus* isolates from milk samples and some of the genotypes were more prevalent. In a previous study [8], it was reported that predominant types of *S. aureus* could be varied in different areas and they may be more resistant to neutrophil bactericidal activities than that of the rare types, which indicates that they may have different features that help them to survive host immunity mechanism.

Several *coa* gene types (547 bp, 680 bp, and 820 bp) were infrequent and only found in a particular location and not present in another location. The infrequent genotypes might be less adapted to the mammary gland therefore easily eliminated from the herd and less easily spread [18]. These different and exclusiveness found among the location may be caused by the pathogen coevolution against its host. The pressure of environments, management, animal trading specific to a certain geographic area would lead to the selection of distinct and genetically adaptable strains [8].

The presence of *coa* genotypes that differ by geographic location and the genotypes that prevailed in each location could be explained by the pathogens-hosts coevolution and the differences in management, nutrition, locations, reservoir bacteria, and environment. The phenomenon of strain homogeneity among some heads in different region also might be explained by the animal trading among regions, the transmission of the strains among herds with close geographic location, the range of discriminatory power of the typing method used, and hosts adaptation to *S. aureus* strains that is present in the environment [18]. The finding of the same genotype among distant location farm could be explained by the interregional herd movement; pathogen spread with human as a carrier, or the homoplasy phenomenon that is the independent acquisition of similar structures without commons ancestors [23].

One isolates analyzed in this study found to produce no amplification products. The isolate showed positive results in *coa* test tube and based on other identification test results phenotypically identified as *S. aureus*. This contradiction results between traditional and molecular method also reported in a former study [24] where 10 strains that classified as *coa* negative by *coa* test tube were found to be positive by a molecular method using PCR. The findings emphasize the use of molecular methods in the identification and detection of *S. aureus* [24,25].

The PCR amplification results of *S. aureus* isolated from cow milk from several herds in East Java, Indonesia, showed the genotype variance of *S. aureus* based on its *coa* gene polymorphism properties. The results also revealed that the *coa* gene types of *S. aureus* strains circulated among herds in the area were varied by 7 types. Among those types, one genotype (510 bp) was predominant, and three genotypes (547 bp, 680 bp, and 820 bp) were unique to particular herds and infrequent. These research results of *coa* gene amplification by PCR are very useful and relatively simple method for *S. aureus* genotyping, and further studies using RFLP technique and sequencing methods on large strains collection from various sources could provide a complete picture of a characteristic of *S. aureus* circulated in the area as well as the epidemiology pattern.

## Conclusion

This study has shown that based on its *coa* gene polymorphism the strains of *S. aureus* that contaminated milk from several dairy farms in three locations in East Java consists of at least 6 types with one genotype predominant strain. It can be concluded that *coa* gene is easily epidemiological tool for detection of variation strain from *S. aureus*. Further researches using RFLP technique and sequencing method on various origin strains might be necessary to understand the epidemiology profile of contamination and infection.

## Authors’ Contributions

MHE is a supervised and project leader. MAMH is a data analysis and collected samples and PH carried out molecular analysis. WT carried out bacterial isolation. All authors contributed in the drafting and revision of the manuscript. All authors read and approved the final manuscript.
